# Archaeal heme *a* synthase: evolutionary trajectory distinct from bacterial homologs

**DOI:** 10.3389/fmicb.2025.1706049

**Published:** 2026-01-02

**Authors:** Val Karavaeva, Marco Rampin, Jordi Zamarreño Beas, Lígia M. Saraiva, Filipa L. Sousa

**Affiliations:** 1 Department of Functional and Evolutionary Ecology, University of Vienna, Vienna, Austria; 2 Vienna Doctoral School of Ecology and Evolution, University of Vienna, Vienna, Austria; 3 Division of Environmental Geosciences, Center for Microbiology and Environmental Systems Science, University of Vienna, Vienna, Austria; 4 Instituto de Tecnologia Química e Biológica António Xavier, Universidade Nova de Lisboa, Oeiras, Portugal

**Keywords:** iron-containing porphyrin compounds, heme biosynthesis, evolution, cofactor, tetrapyrroles

## Abstract

Hemes are iron-containing porphyrin compounds that play a crucial role in a wide array of biological processes. Heme *a* synthase (HAS) is a crucial enzyme that facilitates the biosynthesis of heme *a*, an essential heme variant that serves as a cofactor in heme-copper terminal oxidases and in some acidophilic archaea's quinol reductases, such as the *ba* complex. Depending on the length and presence or absence of cysteine residues in the periplasmic loop(s), HAS has been classified into different types. Our manuscript presents a large-scale analysis of the distribution and evolution of HAS in Archaea in comparison to Bacteria, revealing evolutionary patterns among the proposed subtypes, with types 1 and 2 diverging early. This study also underscores the complexity of HAS enzyme evolution, which reflects deep ancestral innovations as well as recent ecological pressures.

## Introduction

Hemes are iron-containing tetrapyrroles with a broad range of biological functions (Bryant et al., 2020). They serve as cofactors in complexes of the canonical electron transport chain, facilitating electron transfer and contributing to the generation of the proton gradient across the membrane necessary for ATP production (Hägerhäll, 1997; Lancaster, 2002; Crofts and Berry, 1998; Crofts, 2004; Wikstrom, 1977; Saraste et al., 1991; Ferguson-Miller and Babcock, 1996; Michel et al., 1998; Pereira et al., 2001). In addition to electron transfer, heme-containing proteins are involved in other biochemical functions, such as oxygen transport, detoxification of reactive oxygen species, acting as sensors, and synthesis of bioactive molecules (Dailey et al., 2017; Dailey and Medlock, 2022; Girvan and Munro, 2013; Paoli et al., 2004; Shimizu et al., 2019; Huang and Groves, 2018; Guengerich and Yoshimoto, 2018).

**Figure 1:**
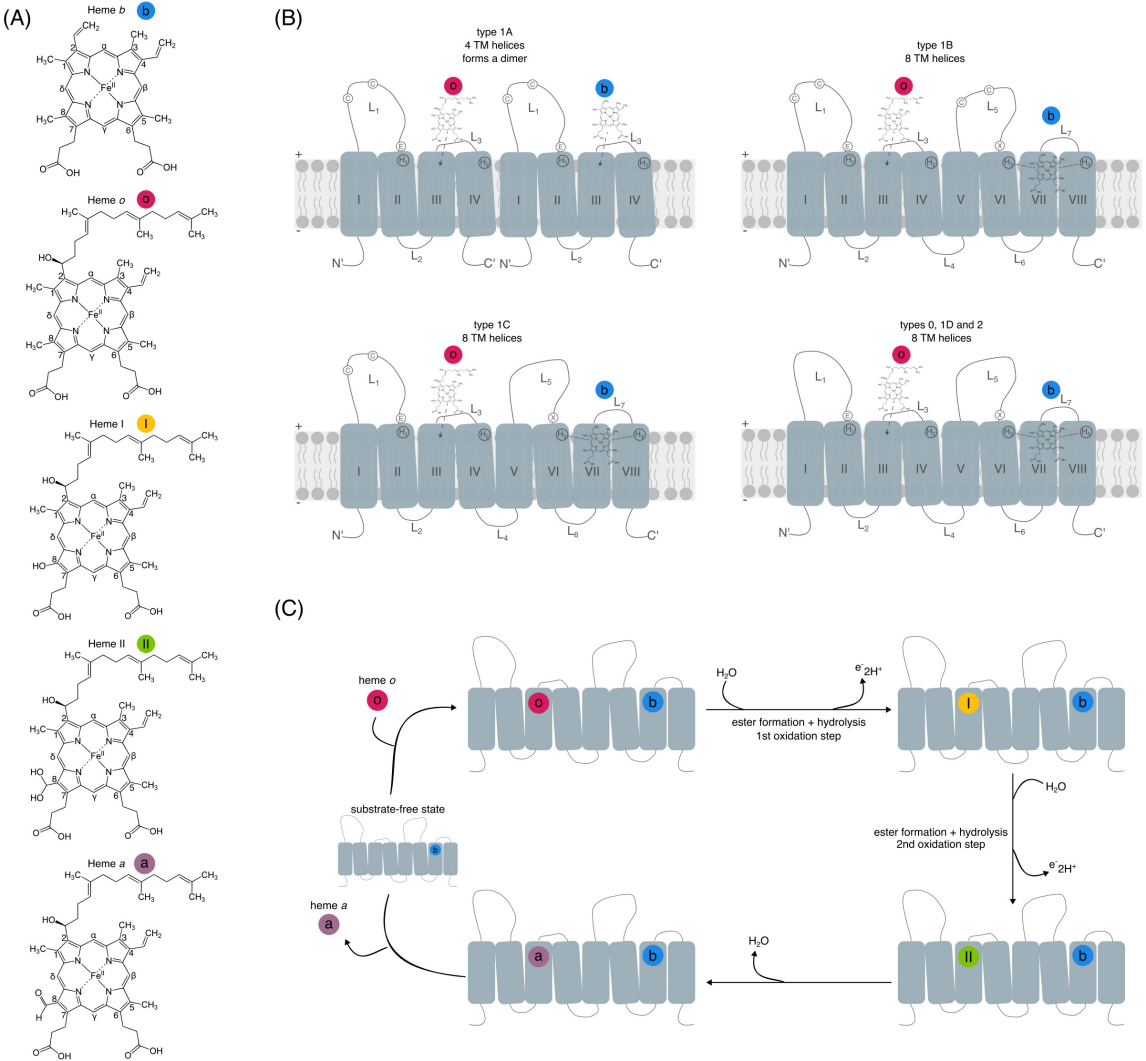
**(A)** Structures of hemes *b, o, a*, and intermediates I and II, labeled according to Fisher nomenclature and color-coded. **(B)** Schematic representation of the canonical HAS types highlighting the number of transmembrane helices (TM; I–VIII) and presence or absence of cysteines **(C)** in the loops (L). H_1_-H_4_ indicate conserved histidine residues, and E denotes the conserved aspartate E57 (*B. subtilis* numbering). X indicates the additional variable catalytic residues equivalent to E57. “+” and “–“ symbols denote the periplasmic and cytoplasmic sides, respectively. Bound heme structures correspond to the initial stage of the reaction. **(C)** A simplified scheme of the proposed HAS reaction mechanism. Adapted Supplementary Figure 7 from Niwa et al. (2018). The hemes and intermediates are color-coded as described in **(B)**.

Hemes *o* and *a* are chemically modified derivatives of the prototypical heme *b* (Figure 1A), synthesized through a sequential enzymatic pathway involving heme *o* synthase (HOS) and heme *a* synthase (HAS; Figure 1B), respectively (Saiki et al., 1992, 1993; Mogi et al., 1994; Svensson et al., 1993; Svensson and Hederstedt, 1994; Svensson et al., 1996; Sakamoto et al., 1999; Barros et al., 2001; Brown et al., 2002, 2004; Hederstedt, 2012; Rivett et al., 2021). Heme *a* has a higher redox potential than hemes *b* and *o*, making it suitable for accepting electrons from donors with higher redox potentials than the ones

of quinones [+95–100 mV for *Caldariella* quinone (Anemüller and Schäfer, 1990), +104–112 mV for ubiquinone (Urban and Klingenberg, 1969)], or cytochrome *c* (Ferguson-Miller and Babcock, 1996; Zhuang et al., 2006; Myer et al., 1979; Tsudzuki and Wilson, 1971). This higher redox potential of heme *a* results from electron-withdrawing properties of the formyl group on pyrrole ring D, which stabilizes the reduced state (Rivett et al., 2021; Figure 1A). Heme *a* serves as a cofactor in (some) type A and type B heme-copper terminal oxidases (Wikstrom, 1977; Saraste et al., 1991; Ferguson-Miller and Babcock, 1996; Michel et al., 1998; García-Horsman et al., 1994; Lübben and Morand, 1994; Lübben et al., 1994; Pereira et al., 2001, 2008) and some acidophilic archaea's quinol reductases, such as the *ba* complex in *Acidianus ambivalens* (Bandeiras et al., 2009; Castelle et al., 2015).

HAS is an integral membrane protein, localized in either the prokaryotic cytoplasmic membrane or the eukaryotic inner mitochondrial membrane (Figure 1B; Glerum and Tzagoloff, 1994; Svensson and Hederstedt, 1994). HAS catalyzes the oxidation of the C8 methyl group on heme *o* (Fisher nomenclature numbering), transforming it sequentially into an aldehyde (Figure 1C; Svensson et al., 1993; Svensson and Hederstedt, 1994; Svensson et al., 1996; Sakamoto et al., 1999; Brown et al., 2002, 2004; Hederstedt, 2012). Early studies showed that HAS requires oxygen for the conversion of heme *o* to heme *a* (Svensson et al., 1996; Sakamoto et al., 1999; Barros et al., 2001; Brown et al., 2002), but later it was shown that the oxygen atom in heme *a*'s formyl group originates from water rather than molecular oxygen (Brown et al., 2004). The exact site of oxygen binding in HAS remains unclear, as both the substrate (heme *o*) and the potential cofactor (heme *b*, which co-purifies with HAS) are capable of binding oxygen (Rivett et al., 2021). In addition, the C-terminal domain of HAS is thought to be the binding-site for a low-spin heme *b* cofactor, whose role in catalysis has not been fully elucidated (Figure 1C). The binding of the heme *o* substrate is thought to be located at the N-terminus (Niwa et al., 2018), where a conserved glutamate (E57 according to *Bacillus subtilis* HAS protein sequence), proposed to be involved in catalysis, is present (Figures 1B, C). Binding of the prosthetic heme *b* and substrate heme *o* is done by four histidine residues: histidine 60 and histidine 123 on transmembrane helix II (TM II) and transmembrane helix IV (TM IV), and histidine 216 and histidine 278 on transmembrane helix VI (TM VI) and transmembrane helix VIII (TM VIII) according to *Bacillus subtilis* numbering. These histidines, which will hereafter be referred to as H1, H2, H3, and H4, respectively, are highly conserved across HAS proteins and considered essential for their activity (Figure 1B; Svensson et al., 1996; Sakamoto et al., 1999; Hederstedt et al., 2005; Mogi, 2009; Hederstedt, 2012; He et al., 2016). Although H1 can be replaced by asparagine as observed through sequence variation across different species in organisms such as A*ctinomycetota* (Mogi, 2009), mutagenesis experiments in organisms naturally possessing a histidine at H1 position have shown that replacing H1 with a leucine resulted in a total loss of activity but did not disrupt the binding of hemes *b* or *o* to HAS (Hederstedt et al., 2005; Hederstedt, 2012). Mutating H2 into leucine or methionine also led to completely inactive proteins (albeit with bound hemes *b* and *o*). This indicates that H1 and H2 may be directly involved in catalysis but not in heme-binding (Hederstedt et al., 2005). Replacing H3 with a leucine disrupted both HAS activity as well as heme-binding, suggesting it is involved in catalysis as well as heme-binding (Hederstedt et al., 2005; Hederstedt, 2012). On the contrary, mutating the terminal H4 did not abolish HAS activity but affected its heme-ligating ability, suggesting that H4 may play a role in heme-binding (Hederstedt et al., 2005; Hederstedt, 2012; Mogi, 2009). Based on these mutagenesis experiments, it has been proposed that H1, H2, and H3 are important for HAS activity, while H3 and H4 serve as heme ligands.

HAS enzymes across all domains of life belong to the Cox15/CtaA family (Mogi et al., 1994; Hederstedt, 2012; He et al., 2016; Rivett et al., 2021). Possibly due to the low sequence similarity among distantly related HAS proteins, HAS homologs have not been identified in the genomes of some Archaea and Eukaryotes, even though these organisms have *aa*_3_ terminal oxidases (He et al., 2016). Most HAS sequences have eight transmembrane helices whilst some have only four, leading to a suggestion that the longer version might have evolved from a duplication and fusion of a shorter version of the protein, which was retained only by some Archaea, e.g., *Aeropyrum pernix* (Lewin and Hederstedt, 2006).

HAS proteins can be divided into two types: type 1, with conserved cysteine residues in the first periplasmic loop, which is found in Archaea and some Bacteria, and type 2, lacking these cysteines and present in Bacteria and Eukaryotes (He et al., 2016; Rivett et al., 2021; named “class D” in Lewin and Hederstedt, 2016). Based on the presence of the cysteine pairs, type 1 can be further separated into at least three subtypes, namely, type 1A (class A in Lewin and Hederstedt, 2016), which is present in some Archaea, with one four-helical bundle that forms functional dimers and maintains a pair of conserved cysteine residues in the first periplasmic loop (L1; Figure 1) (Lewin and Hederstedt, 2006); type 1B (class B in Lewin and Hederstedt, 2016), with a cysteine pair in both L1 and L5 (Figure 1), and type 1C (class C in Lewin in Hederstedt), with a cysteine pair found only in L1 (Lewin and Hederstedt, 2016). The less conserved C-terminal cysteine pair (Cysteine 191, Cysteine 197) is not required for activity, while the N-terminal cysteine pair is important for activity in type 1 HAS (Cysteine 35, Cysteine 42) (Hederstedt, 2012; Lewin and Hederstedt, 2016). Types 1B, 1C, and 2 comprise most of the known HAS sequences and have 8 transmembrane helices (Figure 1). Recently, an updated classification was proposed, introducing type 0 (devoid of cysteine pairs), as well as six additional subtypes of type 1, taking into account the variable lengths of the periplasmic and cytoplasmic loops (referred to as extra- and intracellular loops, respectively, in Degli Esposti et al., 2021). Of those, type 1D (referred to as type 1D in this manuscript from now on) is devoid of cysteines but more closely related to type 1 than to type 2. Table 1 provides an overview of the currently existing HAS classifications, including the one discussed in this manuscript.

**Table 1:** An overview of existing HAS classifications, including the basis for the classification and the original reference.

**Classification**	**This manuscript**	**Lewin and Hederstedt (2016)**	**He et al. (2016)**	**Degli Esposti et al. (2021)**
Basis	Conserved cysteines, number of transmembrane helices, and phylogenies	Conserved cysteines and the number of transmembrane helices	Conserved cysteines and phylogenies	Conserved cysteines, number of transmembrane helices, and periplasmatic loop length
Types	Type 0	Class D	-	Type 0
	Type 1A	Class A	Type 1	Type 1.4
	Type 1A^*^	Class C	-	-
	Type 1B	Class B	Type 1	Types 1.1 and 1.2
	Type 1C	Class C	Type 1	Types 1.0 and 1.3
	Type 1D	Class D	Type 1	Type 1.5
	Type 2	Class D	Type 2	Type 2.0
Reference	This manuscript	https://doi.org/10.1016/j.bbabio.2015.11.008	https://doi.org/10.1093/molbev/msv201	https://doi.org/10.3389/fmicb.2021.664216

Despite advances in the understanding of HAS structure and its catalytic mechanism, the distribution and evolution of different types of HAS enzymes have not been studied on a larger scale, and both the current proposed classifications and the existing evolutionary scenarios relied on rather small datasets, i.e., 127 sequences (He et al., 2016) and 60 sequences (Degli Esposti et al., 2021). It is therefore unclear whether any of the current classifications or evolutionary scenarios would hold in the context of evolution on a larger scale. Specifically, it is unclear whether the short form of HAS found in Archaea represents an ancestral form of the enzyme, as proposed in Lewin and Hederstedt (2006), or if, instead, a domain of unknown function with 4 TM helices could be at the root of this family of enzymes (Degli Esposti et al., 2021). Thus, these uncertainties, together with the limited datasets used, render the conclusions about the origins of HAS inconclusive. This manuscript aims to fill this gap by reporting on the taxonomic distribution of different types of HAS in Archaea relative to Bacteria, using a comprehensive dataset of over 35,000 genomes, and by proposing a revised evolutionary scenario for the evolution of these enzymes, as well as addressing the pitfalls of the continuous (re-)classification of the HAS family.

## Materials and methods

### Query gathering and pre-processing

The enzymes responsible for synthesizing heme *a* were obtained through a review of scientific literature (Rivett et al., 2021), resulting in the identification of five characterized HAS sequences (HAS, EC 1.17.99.9). These were downloaded from the BRENDA database (Chang et al., 2021) (Supplementary Table 1) and clustered using the CD-HIT (Li and Godzik, 2006) tool in Galaxy Pasteur (Mareuil et al., 2017) at a similarity cut-off of 0.9, though each sequence formed its own cluster. The sequences were then aligned using Clustal Omega (version 1.2.4, Sievers and Higgins, 2018) and visualized with Jalview (version 2.11.3.3, Waterhouse et al., 2009) to confirm homology, identify conserved regions, and determine HAS types based on the presence or absence of conserved cysteine pairs (Niwa et al., 2018; Rivett et al., 2021). Since type 0 exhibits low identity to other types and cannot be found by standard searches (Degli Esposti et al., 2021), we used two type 0 sequences as queries (HAS type 0 from *Acidithiobacillus ferrooxidans ATCC 23270* and from *Sulfolobus metallicus*, as listed in Degli Esposti et al., 2021). In total, 7 query sequences, representing all canonical types, as well as non-canonical type 0, were gathered.

### Initial hits and filtering

The query sequences were searched against an in-house genomic database (35,307 assemblies, Supplementary Table 2) using Diamond BLAST (version 2.1.9, Buchfink et al., 2021). Completeness and redundancy of the in-house dataset assemblies were computed using the “Rinke method” (Rinke et al., 2013). Hits were filtered for higher than 25% sequence identity and an E-value below or equal to 10^−10^. The remaining sequences were globally aligned using the Needleman-Wunsch algorithm (Needleman and Wunsch, 1970) in the EMBOSS package (version 6.6.0.0, Rice et al., 2000). Sequences with over 25% global identity were retained for further analysis.

To refine the dataset and eliminate false positives, clustering was performed with the Markov clustering algorithm (van Dongen, 2008) via the Galaxy platform (Galaxy Community, 2024). Clusters with an Identity greater than 25%—based on mean and median inter-cluster comparisons—were merged and analyzed as a single group. This is the canonical cut-off for homology detection. Sequence reduction was performed using a 90% sequence identity cut-off.

**Figure 2:**
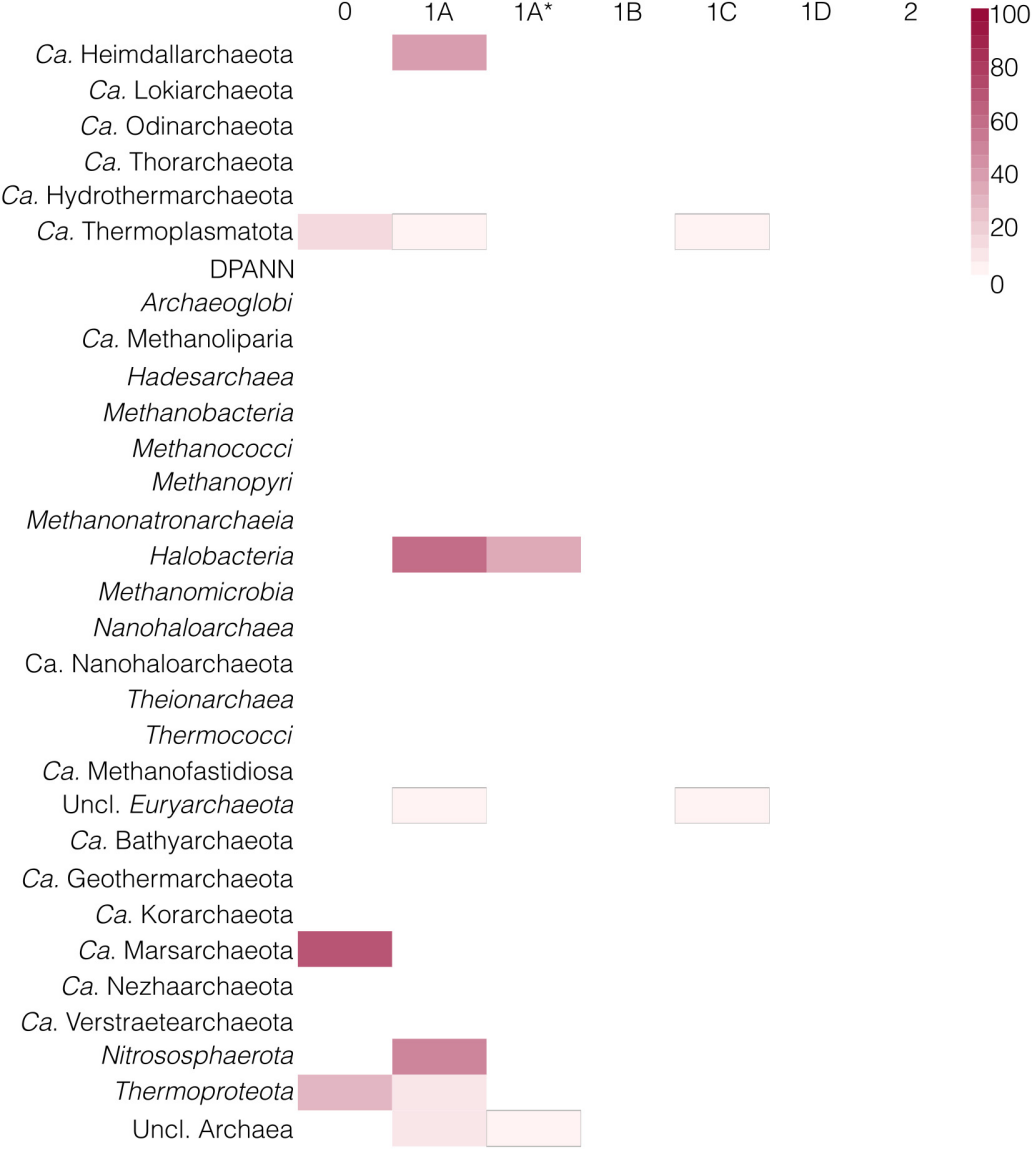
Taxonomic distribution of different types of HAS in Archaea, sorted by NCBI taxonomy. White color indicates the absence of a certain type. Red color indicates the presence of a certain type in a certain lineage; the darker the color, the higher the percentage of genomes from a lineage that have a hit to the specific type of HAS. *Ca*. is an abbreviation of “*Candidatus*.” To aid in the visualization of low-abundant HAS types in a lineage, those are delimited with black outlines.

In addition, all sequences listed in the CtaA phylogeny (Figure 2 from Degli Esposti et al. (2021), as well as sequences belonging to DUF420 from Degli Esposti et al. (2021), were retrieved and added to our analysis (Supplementary Table 3).

### Multiple sequence alignment and phylogenetic reconstruction

The filtered sequences were used to construct a multiple sequence alignment using Clustal Omega, with 100 iterations for both the guide tree and HMM. Alignments were visualized in Jalview and examined for quality control. The alignments were subsequently trimmed using trimAl (version 1.5.0, Capella-Gutiérrez et al., 2009) with a 60% conservation threshold and a 0.05 -gt threshold. Trimmed alignments were reviewed in Jalview to confirm appropriate fusion cutting and gap removal. Fusion proteins, i.e., the ones in which an HAS domain was fused to another gene, were not included in the alignments. Maximum likelihood phylogenetic reconstructions were generated using IQ-TREE (version 2.3.6, Minh et al., 2020) with ModelFinder (Kalyaanamoorthy et al., 2017) and 1,000 ultrafast bootstrap iterations (Hoang et al., 2018). In addition to a phylogeny including all HAS types, a phylogeny was also computed without the type 1A and 1A^*^ to assess whether these types could interfere with the alignment, as stated in Degli Esposti et al. (2021). Moreover, a structural phylogenetic analysis was performed using the method described in Garg and Hochberg (2025). All available Alphafold predicted structural models (Jumper et al., 2021; Varadi et al., 2021) for HAS sequences were obtained using the 3D Beacons HUB API (Varadi et al., 2022). 3Di information was extracted from these models using Foldseek (van Kempen et al., 2023). The resulting 3Di sequences were aligned using Mafft (version 7.525; Katoh et al., 2002; Katoh and Standley, 2013; Nakamura et al., 2018) with the *ginsi* algorithm and the –aamatrix option, employing the 3Di scoring matrix of Foldseek (van Kempen et al., 2023). The resulting alignment was trimmed as previously described, and a maximum likelihood phylogeny was constructed using IQ-TREE (version 2.3.6, Minh et al., 2020) with the custom model Q.3Di.AF (Garg and Hochberg, 2025), and 10,000 ultrafast bootstrap iterations (Hoang et al., 2018).

Maximum-likelihood phylogenies were rooted using the minimal ancestor deviation method (MAD; version 2.22, Tria et al., 2017). FigTree (version 1.4.4, Rambaut, 2009) was used to export an SVG file, which was subsequently beautified with Inkscape (version 1.3.2, The Inkscape Project, 2024). All phylogenetic reconstructions were further annotated, including the sequence's HAS type, functional annotation, taxonomy, conserved motifs, lengths of the periplasmic loops, and syntenic information. Phylogenies in the Newick format and their respective annotation table are available at Figshare: 10.6084/m9.figshare.30041233.

### Functional annotations

The query sequences and their obtained homologs were functionally annotated using KEGG profiles (version 2024-02-28; Kanehisa and Goto, 2000) by running HMMER (version 3.4; hmmer.org) and filtering the resulting hits by provided model thresholds (file “ko_list” in KEGG). In addition, we ran Interproscan (version 5.74-105.0-11.0.4; Jones et al., 2014) for an expanded functional annotation, as this tool contains multiple databases relevant to our analysis, such as CDD (NCBI Conserved Domain Database; Lu et al., 2020), NCBIfam (Li et al., 2021), PANTHER (Thomas et al., 2022), PFAM (Mistry et al., 2007), and SUPERFAMILY (Gough and Chothia, 2002; Wilson et al., 2009). Since HAS is a transmembrane protein, we also ran TMHMM (version 2.0; Krogh et al., 2001; Sonnhammer et al., 1998) to predict the number of transmembrane helices. In addition, the loop length of periplasmic loop 1 (ECL1; L1 in Figure 1) and periplasmic loop 3 (ECL3; L5 in Figure 1) (using the numbering from Degli Esposti et al., 2021) was calculated using the results from TMHMM and plotted using the ggplot package in R (https://cloud.r-project.org/web/packages/ggplot2/index.html; R Core Team, 2024; Wickham, 2016). The full functional annotation table is available as Supplementary Table 4.

### HAS type classification and taxonomic distribution

HAS sequences were classified into types 0, 1 (A, B, C, D), and 2 according to the presence or absence of conserved cysteine residues. The classification was later verified by their placement in the phylogeny. Partial sequences (less than 100 amino acids) were not assigned to any HAS type. Mean, median, maximum, and minimum global and local identities were computed between the different HAS types as well as the DUF420 representative sequences. The taxonomic distribution of these types was calculated per genome (Supplementary Table 5) and per phylum (or class in case of *Euryarchaeota)*. DPANN archaea and bacterial Candidate phyla with no hits were collapsed into one category to simplify data visualization in the final plots. The resulting matrix per phylum for Archaea and Bacteria was plotted as heatmaps using the R package pheatmap (https://cran.r-project.org/web/packages/pheatmap/pheatmap.pdf).

### Synteny

The syntenic neighborhood of identified HAS sequences was calculated using a window of 5 upstream and 5 downstream neighboring sequences. These neighbors were functionally annotated and analyzed for conservation patterns, using HAS type notation for HAS sequences, and KO for neighbors (or PFAM/SUPERFAMILY if no KO had been assigned to the neighbor sequence). The resulting syntenic blocks were mapped to phylogenies for subsequent analysis.

## Results

Our analysis identified a total of 8,093 potential HAS sequences, divided into 12 distinct clusters (three containing query sequences). Based on the presence or absence of the two cysteine pairs, sequences were classified as type 1A (273 sequences), type 1B (2,339 sequences), type 1C (924 sequences), and type 2 (4,321 sequences), respectively. Types 1D and 0 (42 and 73 sequences, respectively; as described by Degli Esposti et al., 2021) were also identified. The remaining types 1.0–1.4, proposed by Degli Esposti et al. (2021) based on loop length, were not considered. In addition, some organisms possess a longer variant (~280 amino acids) of the presumed type 1A HAS, containing eight transmembrane helices. To our knowledge, this form has not been previously described in Archaea, and we therefore designated it as type 1A^*^ (121 sequences). It is unclear whether these type 1A^*^ sequences constitute a *bona fide* HAS, as this type has not previously been described, to the best of our knowledge. Similarly, whether type 0 and type 1D constitute a *bona fide* HAS is also unclear, since no biochemical characterization of these enzymes has been conducted, with only comparative genomics and transcriptomics studies available (Appia-Ayme et al., 1999; Quatrini et al., 2009; Acuña et al., 2013; Issotta et al., 2018; Degli Esposti et al., 2021). While type 1A^*^, type 1D, and type 0 sequences may plausibly encode HAS, given their conserved residues, structural features, and synteny, this remains an inference.

### Taxonomic distribution of HAS types in Archaea vs. Bacteria

Out of 24 archaeal phyla, only 7 possess a HAS enzyme: *Candidatus (Ca.)* Heimdallarchaeota, *Euryarchaeota* (*Halobacteria* and unclassified *Euryarchaeota), Ca*. Marsarchaeota, *Ca*. Thermoplasmatota, *Nitrosophaerota, Thermoproteota*, as well as unclassified Archaea (Figure 2). Most of these archaeal phyla have type 1A HAS, with some *Halobacteria* and unclassified Archaea having a longer homologous protein, herein named type 1A^*^. Many of type 1A HAS sequences are fused to heme *o* synthase (154/273–56%), with most of these fusions occurring in *Halobacteria*. Although it is unclear whether type 1A^*^ is a *bona fide* HAS, most of these sequences (111/121–92%) are the only putative “HAS” found in the genome, with only 10 cases co-occurring with a HAS of type 1A in the same assembly. Moreover, the presence of heme *a*-dependent proteins in these lineages (Sorokin et al., 2018), some of which have been experimentally characterized and shown to contain heme *a* (Kanti Das et al., 2004; Wakagi et al., 1989; Bandeiras et al., 2009), might indicate that type 1A^*^ proteins are indeed HAS. Moreover, heme-binding redox sensors were discovered within *Halobacteria* (Hou et al., 2001), and although by their UV-VIS spectral signatures they contained heme *b*, these sensors could in some species harbor heme *a*. Interestingly, some *Ca*. Thermoplasmatota and unclassified *Euryarchaeota* have a HAS of type 1C (Figure 1), which has not been previously reported in Archaea. Type 0 is found in *Thermoproteota, Ca*. Marsarchaeota and *Ca*. Thermoplasmatota. Types 1D (which has no conserved cysteines but is clustered within type 1), 1B, and 2 are absent in Archaea. Only in *Ca*. Marsarchaeota all assemblies contained a HAS enzyme, indicating the low usage of heme *a* in the archaeal domain. In fact, with the exception of complex IV and the *ba* complex (e.g., *Acidianus ambivalens*, as reported in Bandeiras et al., 2009), no other enzyme is known to use this heme type in Archaea. Notably, all *Acidianus* genomes in our dataset contain a type 0 hit, which is also present in 76% (32/42, including 7/7 *Acidianus* genomes) of our *Sulfolobales* genomes. Of note, in 10 *Sulfolobales* assemblies, no type 0 was found, possibly due to their incompleteness.

**Figure 3:**
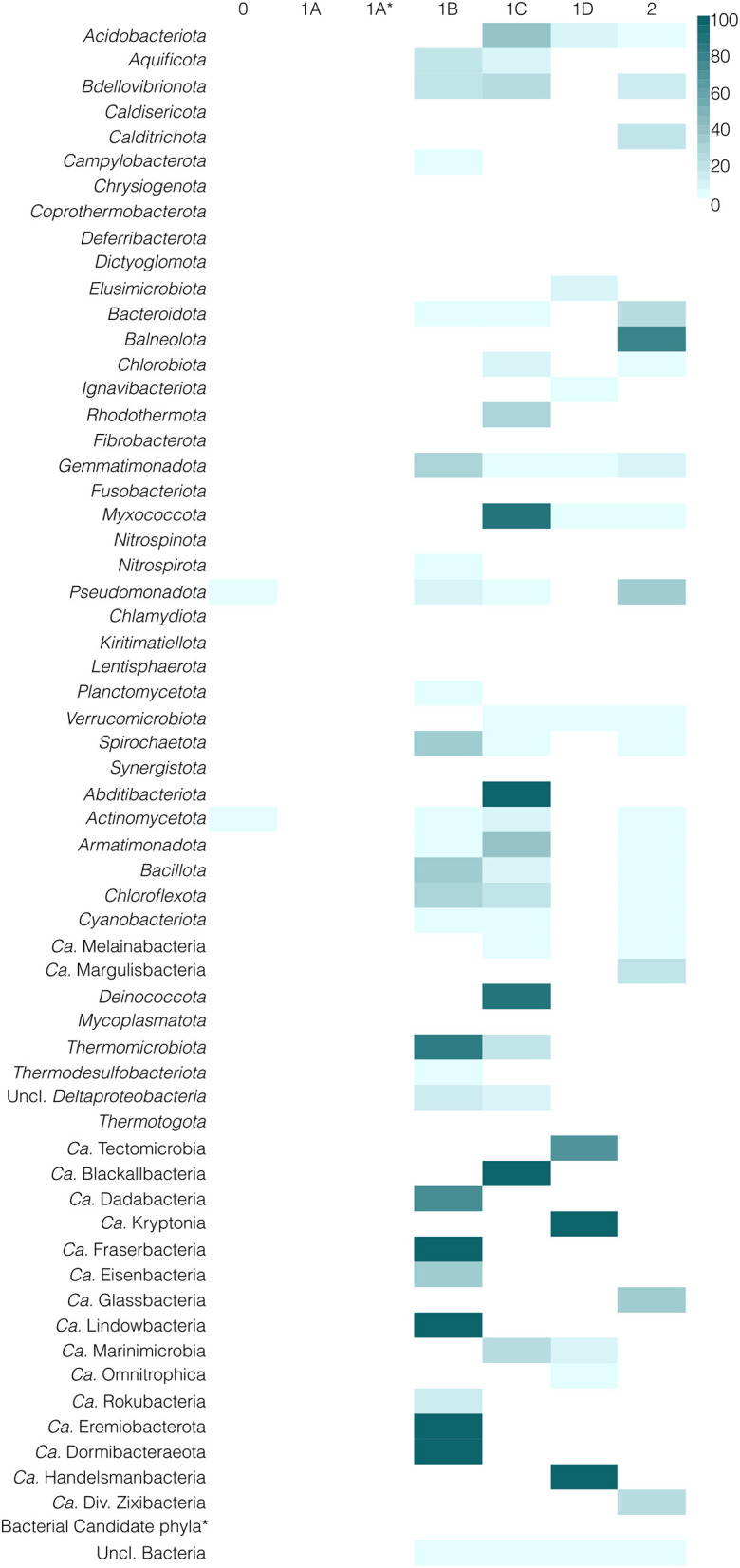
Taxonomic distribution of different types of HAS in Bacteria, sorted by NCBI taxonomy. White color indicates the absence of a certain type. Blue color indicates the presence of a certain type in a certain lineage; the darker the color, the higher the percentage of genomes from a lineage that have a hit to the specific type of HAS. *Ca*. is an abbreviation of “*Candidatus*.” ^*^indicates phyla without HAS.

In contrast, type 1A and type 1A^*^ are completely absent from Bacteria (Figure 3). Instead, the other types are widespread, with type 1B present in 25 phyla, type 1C in 24 phyla, type 2 in 21 phyla, and type 1D found in 12 phyla. Some bacterial lineages, e.g., *Bdellovibrionota, Bacillota, Spirochaetota*, have a combination of two or even all three of these types. Conversely, type 0 is only present in some *Pseudomonadota* and *Actinomycetota*.

The observed taxonomic distribution suggests that HAS proteins in Archaea and Bacteria are divergent since the presence of nearly all HAS types (except type 0) is restricted to either one or the other domain. However, this does not clarify their functional or evolutionary relationships. To address these questions, we conducted a synteny analysis and constructed maximum-likelihood phylogenetic reconstructions of HAS enzymes.

The mean and median identity between HAS types, as well as with DUF420, were computed by using both local and global identities (Supplementary Table 8). Using global identities, the intertype HAS mean and median identity values are low, but above 20. However, their relationships with DUF420 fall in the ranges below 15, except for types 1A and 1B, where maximum values of around 20 and 22 are obtained, with mean and median values still below 15. Of note, with local identities, the relationship between this domain of unknown function with the different HAS types could not be computed due to a lack of significant similarity (internal Diamond cut-offs). These results prompt the exclusion of DUF420 from phylogenetic analyses and lead us to question its homology with this family of enzymes.

### Syntenic neighborhood of HAS

We examined the genomic neighborhoods of HAS, as described in the Methods, and identified recurring patterns of conserved synteny (Supplementary Table 6). A clear set of associations emerges, highlighting both shared metabolic connections and divergent evolutionary trajectories among HAS types. Heme *o* synthase (HOS) is most frequently found with HAS types 1B, 1C, and 1D, and shows less co-occurrence with type 0 (2,140/2,339 (91.5%), 695/924 (75.2%), 37/42 (88.1%), and 12/73 (16.4%) cases, respectively). Given its role in the maturation of heme cofactors, this strong syntenic association of HOS and HAS suggests that these HAS types are regulated in direct connection with respiratory electron transfer pathways, where heme *o* or heme *a* incorporation is critical. Its near-total co-occurrence absence from types 1A/1A^*^ and its rarity in type 2 neighborhood imply that these forms of HAS are uncoupled from the expression of cytochrome/HCO-mediated respiration and have a different regulatory mechanism. In addition, cytochrome *c* oxidase subunits show a complementary distribution, typically co-localizing with HAS types 1A, 1B, 1D, and 0 (96/273 (35.2%), 1,371/2,339 (58.6%), 33/42 (78.6%), and 18/73 (24.7%) cases, respectively). Their scarcity with type 1C and type 2 (112/924 (12.1%) and 31/4,321 (0.7%) cases, respectively) echoes the observation above, reinforcing that types 1B and 1D (and to some degree type 0) represent a respiratory-specialized cluster, while type 2 has diverged from these trajectories. HAS of types 1B and 1C also share conserved synteny with DUF1507 proteins, pyruvate carboxylase, and the cell division protein FtsW. The synteny with pyruvate carboxylase might point to coupling of HCOs with central carbon metabolism (Lai et al., 2006; Mukhopadhyay et al., 2000; Payne and Morris, 1969; Jitrapakdee and Wallace, 1999; Attwood, 1995), while the synteny with FtsW suggests an intersection with cell envelope biogenesis and division (Käshammer et al., 2023; Mohammadi et al., 2011; Taguchi et al., 2019). The repeated presence of DUF1507 indicates a yet-uncharacterized but functionally linked role, likely unique to organisms containing this HAS type. The conserved synteny between HAS types 1B, 1D, and 2 and SCO1—a known copper-trafficking factor essential for cytochrome *c* oxidase assembly (Petruzzella et al., 1998)—reinforces the respiratory-linked regulation of HAS in these lineages, though this association is less robust for type 2. In contrast, HAS type 2 displays a markedly different genomic context. Its neighbors are dominated by ribosomal proteins (S9 and L13), enzymes of amino acid metabolism (N-acetyl-γ-glutamyl-phosphate reductase and agmatinase), acetyltransferases (GNAT domain), and chain length determinant proteins. The proximity with these housekeeping proteins suggests that type 2 HAS is embedded within a more generalized biosynthetic operon environment, possibly being constitutively expressed. Type 0 HAS is also flanked by distinctive neighboring genes, such as tRNA(Met) cytidine acetyltransferase, γ-glutamyltranspeptidase, MazG-domain nucleotide pyrophosphohydrolases, and BadF/BadG/BcrA/BcrD ATPases. Many of these genes are implicated in stress response, detoxification, or specialized metabolic regulation, suggesting that type 0 HAS may be expressed during adaptive or auxiliary redox processes under fluctuating physiological conditions.

Types 1A and 1A^*^ reveal limited overlap in terms of syntenic partners, sharing only malate dehydrogenase, a central enzyme of the TCA cycle, and polar amino acid transporters as genes in their proximity. Type 1A often clusters with selenoprotein W-related protein genes, archaea-specific RecJ-like exonucleases, and FAD synthetases—pointing toward regulatory integration and possible links to secondary metabolism. In contrast, type 1A^*^ is associated with DUF5814 proteins, replication factor C (replication clamp loader), and binding-protein-dependent transport systems, indicating that its regulation might be a connection to cell cycle progression and nutrient uptake control.

These patterns point to two broad evolutionary and regulatory groupings. HAS types 1B, 1C, and 1D are consistently associated with respiratory chain components and central metabolic enzymes, suggesting coupled expression patterns. In contrast, types 0, 1A, 1A^*^, and especially type 2 appear to have diverged into more diverse genomic contexts—sometimes associated with stress response, informational processing, and translational machinery, or secondary metabolism—indicating the potential decoupling of direct expression with heme *a*-containing HCOs or a synchronized albeit separated expression pattern. This division likely reflects adaptation of HAS regulation into distinct cellular roles, with some types remaining directly coupled with aerobic respiration and others expanding into more varied regulatory networks.

### Phylogenetic reconstruction of HAS

Multiple sequence alignments were performed by selecting all HAS enzyme types as well as by excluding types 1A and 1A^*^ from one of the alignments to see if these sequences disrupted the alignment, as stated by Degli Esposti et al. (2021). Those were inspected for conservation of important residues and quality of the alignment. Among the first conserved motif in helix 2, the glutamate, proposed to be important for function (Niwa et al., 2018), is strictly conserved. The motif can be represented as E57–X1–X2–H1–R, where X1 and X2 vary between types. In position X1, a tryptophan is usually found in types 1A (85%), 1A^*^ (96%), and 2 (74%). This position is occupied by a histidine in type 1D (98%), a phenylalanine or a tyrosine in type 1C (60% and 25%, respectively), and an alanine in type 0 (75%). In type 1B, the position X1 is not conserved. The position X2 is highly variable across most of the HAS types, with the exception of type 1A^*^, where a phenylalanine is found in 87% of the cases. The H1 position is conserved across all types, being replaced by an asparagine in 34% of type 1C sequences, in agreement with previous reports (Mogi 2009). The “mirror” motif at helix 6, X1–X2–X3–H3–X4, is in general much less conserved. In type 0, position X1 is most frequently occupied by a tryptophan (55%) or a phenylalanine (15%) residue, while positions X2, X3, and X4 are highly variable. The mirror motif is absent in type 1A due to the sequences being truncated at the C-termini. In type 1A^*^, histidine H3 is replaced by a phenylalanine in 55% cases. Of note, type 1A^*^ has a conserved glutamine at X1 (93%), a partially conserved alanine at X2 (59%), and a tyrosine at X4 (78%). There is no observed amino acid conservation at the X3 position. All remaining types (1B, 1C, 1D, and 2) have no conserved residues at X3, exhibit some conservation at X1 (glutamine in 1B and 2, arginine in 1C, and histidine in 1D), and, except for type 1C, have an arginine at X4. Types 1D and 2 have a conserved phenylalanine at position X2 (63% and 91%, respectively), with types 1B and 1C showing no conservation at this position. The natural variability observed at positions H1 and H3 suggests distinct roles in heme binding. Position H1 consistently contains either histidine or asparagine residues, both of which are capable of binding heme. In contrast, not all of the residues found at position H3 are compatible with heme binding, indicating that H3 is unlikely to participate in this function, at least in 55% of the type 1A^*^ sequences.

**Figure 4:**
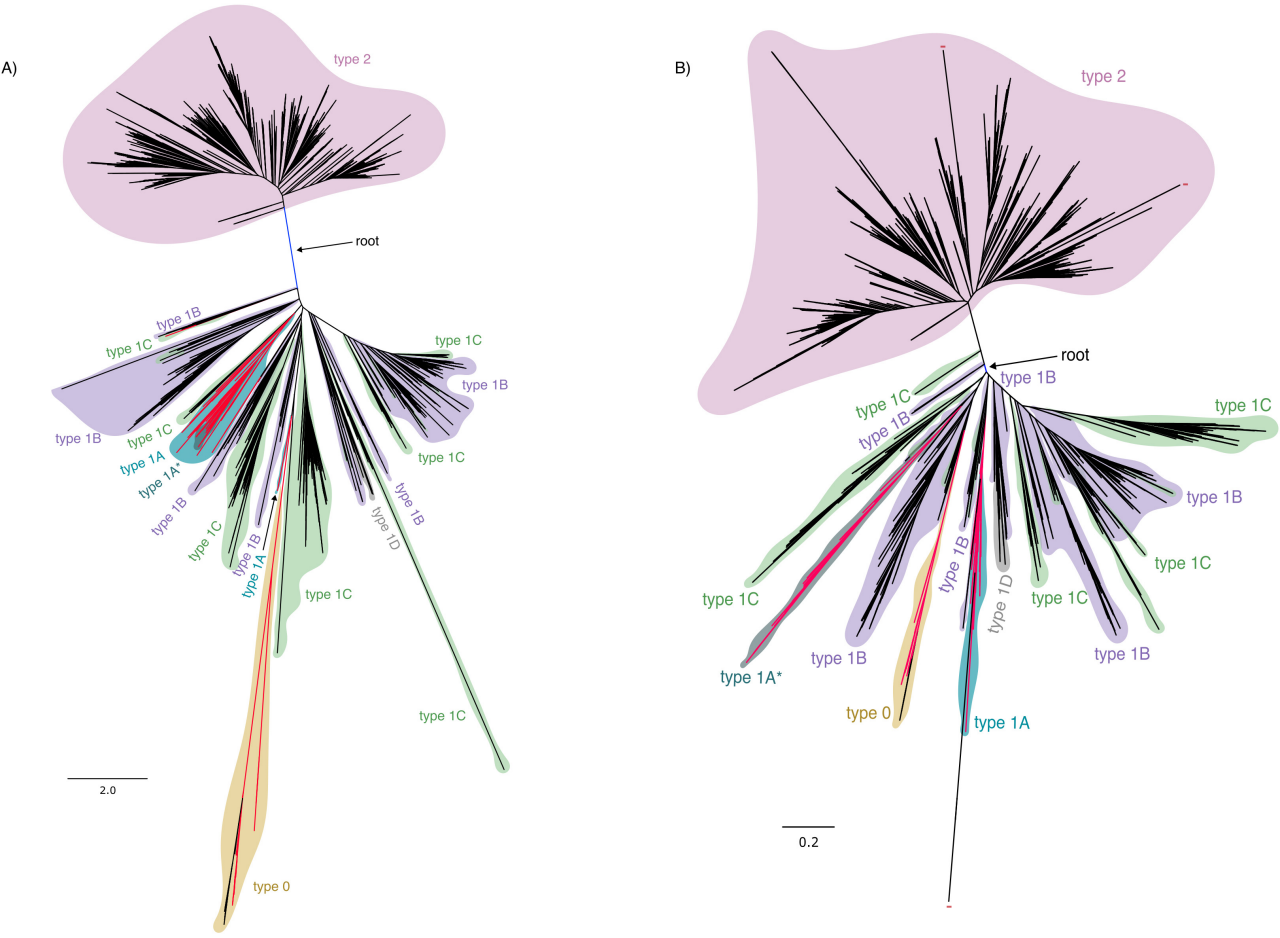
**(A)** Maximum-likelihood phylogenetic reconstruction of HAS based on an amino acid multiple sequence alignment (Best-fit model: Q.Pfam+F+R10), colored as follows: type 0 is yellow, type 1A is cyan, type 1A* is dark turquoise, type 1B is purple, type 1C is green, type 1D is gray, and type 2 is pink. Root is marked in blue. Red branches indicate archaeal sequences, black indicate bacterial sequences. The scale bars indicate the corresponding number of nucleotide substitutions per site. **(B)** Maximum-likelihood phylogenetic reconstruction of HAS based on the 3Di structural alignment (Custom model: Q.3Di.AF), colored as follows: type 0 is yellow, type 1A is cyan, type 1A* is dark turquoise, type 1B is purple, type 1C is green, type 1D is gray, and type 2 is pink. Root is marked in blue. Red branches indicate archaeal sequences, black indicate bacterial sequences. The scale bars indicate the corresponding number of structural differences per site.

The maximum likelihood phylogenetic reconstruction of all HAS types is shown in Figure 4A (for original phylogenies in the Newick format, see additional data at Figshare). In addition, due to the low conservation of membrane proteins, we also performed a structural phylogeny with a subset of our data using the strategy from Garg and Hochberg 2025 (Figure 4B and additional data at Figshare). To assess if the inclusion of types 1A and 1A^*^ affected the alignment, a maximum-likelihood (ML) reconstruction without these types of enzymes was performed (Supplementary Figure 1 and additional data at Figshare). The overall topology of the phylogenies is similar, with the root separating type 2 HAS from the remaining enzyme types. This separation is in agreement with what had been previously observed in He et al. (2016) and Degli Esposti et al. (2021). The main difference is seen in the structural phylogeny, where type 1A^*^ is in a separate clade from type 1A.

*Pseudomonadota* sequences are basal on both sides of the root (specifically, class *Alphaproteobacteria*) and the most abundant lineage in the type 2 clade. On the other side of the root, type 1B sequences are basal to the other types; however, this type is not monophyletic. Both type 1B and type 1C are extensively intercalated in the phylogeny. Notably, two archaeal sequences of type 1C stem out of the basal type 1B clade, which itself is composed mostly of *Pseudomonadota* sequences. Even within individual phyla, e.g., *Bacillota*, several type 1C subclades are observed, each with type 1B sequences at their base. This pattern indicates that cysteine loss occurred multiple times independently, even within a single phylum, and that the current classification does not reflect the evolutionary origin or evolutionary events of the HAS family. On the other reconstructions, with the exception of the structural phylogeny, most of the type 1A and 1A^*^ sequences are in the same clade, with type 1A basal to 1A^*^. This suggests that type 1A^*^ may represent a fusion or extension of 1A. However, exceptions exist: two type 1A sequences from the genus *Aeropyrum* cluster separately, branching from a type 1C clade and appearing basal to type 0. In addition, a type 1A sequence from unclassified Archaea and one from unclassified *Nitrososphaerota* form a separate clade stemming from within the type 1B group. Types 1D and 0 are monophyletic. Type 1D is exclusively found in Bacteria and stems from within a type 1B clade. Type 0 stems out of a small type 1A clade, which consists exclusively of *Aeropyrum* sequences. Type 0 clade has archaeal sequences as basal, followed by several *Pseudomonadota* and *Actinomycetota* sequences that acquired this type via lateral gene transfer (LGT). None of the archaeal basal type 0 sequences belong to acidophilic organisms (*Thermoproteus uzoniensis, Pyrobaculum ferrireducens, Pyrobaculum calidifontis)*. The long branch of this type can reflect its potential for fast evolution and reflects the overall low identity to the other HAS types.

Generally, there is a distinction between types 1 and 2 of HAS. While type 2 is represented by a single large monophyletic clade, type 1 exhibits internal complexity, where types 0 and 1D constitute “special cases,” and types 1A and 1A^*^ likely result from several LGTs coupled to a fission event, with type 1A also being non-monophyletic. Also, types 1B and 1C are not monophyletic either, with type 1C evolving from type 1B multiple times. Therefore, the current classification is decoupled from the evolutionary events of this protein family.

We analyzed the clades of different types in relation to lengths of the periplasmic loops ECL1 and ECL3 (numbering from Degli Esposti et al., 2021; correspond to L1 and L5 in Figure 1, respectively), and found that these lengths vary not only within the same type and lineage—on both phylum and class level—but also within smaller intercalated clades, particularly for types 1B and 1C (see phylogenies and their annotations at Figshare, as well as Supplementary Table 7 and Supplementary Figure 2). The loop lengths tend to be longer on average in type 2 than in other types of HAS, with those in type 0 being generally shorter. However, no consistent relationship pattern has emerged between the clade position of sequences and their corresponding loop lengths. This observation provides no support for further classification of types 1B and 1C into subtypes based on loop lengths.

Additionally, we have mapped the motifs E57–X1–X2–H1–R and X1–X2–X3–H3–X4 onto the phylogenies to discern whether there is a connection between these motifs and intercalated clades of types 1B and 1C (see phylogenies and their annotations at Figshare). We observed that, similarly to the loop lengths, there is a variability of motif patterns within the same clade for all HAS types. Rather than being type-specific, the motif conservation seems to reflect the taxonomic conservation on the genus level.

## Discussion

In this study, we have performed a large-scale analysis of HAS across more than 35,000 genomes, integrating taxonomic distribution analyses and the classification based on the conservation of the cysteine pairs, with sequence-based and structure-based phylogenetics. We have identified 8,093 HAS distributed in 52 phyla (45 bacterial and 7 archaeal). These could be classified into 7 different types, from which types 2 and 1B are found exclusively in Bacteria, while type 1A and a potential novel 1A^*^ are found exclusively in Archaea. The possible identification of a novel archaeal type (1A^*^−121 sequences), found in *Halobacteria* and unclassified *Euryarchaeota*, with 8 TM helices and whose position in the phylogeny is still to be clearly determined, further highlights the diversity of this family of enzymes. If type 2 is restricted mainly to *Pseudomonadota* and *Bacillota*, type 1 HAS shows a much larger taxonomic and structural conservation variability. These observations might support the view that the evolution of HAS reflects both deep ancestral innovations and more recent ecological pressures.

Our results provide new insights into the diversity, evolutionary history, and structural conservation of this key enzyme in aerobic respiration and re-examine the validity of the existing classifications. The broad distribution of HAS types across bacterial and archaeal lineages might suggest that the diversification of this enzyme family occurred early in the evolution of aerobic metabolism. While some lineages retain only one HAS type, others show clear signs of divergence, with more than 1 type of HAS per assembly, including clade-specific expansions and possible losses. The patchy presence of HAS in certain taxa is consistent with scenarios of LGT and lineage-specific adaptation, particularly in organisms occupying environments with variable oxygen availability. These could be the cases of, e.g., type 2 enzymes in *Gemmatimonadota, Verrucomicrobiota*, and *Ca*. Margulisbacteria, as well as the two type 1C sequences found in Archaea.

Our phylogenetic analyses based on sequence alignments largely reproduced the expected type and taxonomic topological relationships, but also revealed incongruencies that point toward a more complex evolutionary history, including potential LGTs, fissions (type 1A), and independent losses of cysteine pairs within the same taxa. This complexity is further highlighted at the level of sequence conservation, in the balance between conservation and divergence. Although the key catalytic residues (histidines and a glutamate) and the overall fold remain conserved across most of the HAS types, lineage-specific variations (at the genus level) of the motifs were identified as well. These may reflect functional specialization, possibly linked to the co-evolution of HAS with terminal oxidases or other respiratory components. In particular, deviations in some archaeal and bacterial lineages raise intriguing questions about potential differences in enzyme activity or substrate interaction. It is possible that, as in Archaea, further heme modifications exist in the bacterial domain, or that the kinetic activities of the enzymes are affected by loop size (Nestl and Hauer, 2014; Corbella et al., 2023). We have observed variability of loop lengths between different HAS types, as well as within the same type, and even within the same clade. Therefore, it does not seem feasible to utilize these loops as a criterion to classify types 1B and 1C into subtypes.

The most parsimonious explanation for the evolution of this family of enzymes would be that type 1A (4 TM) or an analogous 4 TM enzyme would be the ancestral HAS form that, after duplication and fusion, would have given rise to the ancestor of type 1 and type 2 enzymes. This idea is proposed in a recent publication (Degli Esposti et al., 2021), using DUF420 (with 4 TMs) as the ancestral enzyme. However, the inclusion of DUF420 in the phylogenetic analysis to root the phylogeny is problematic. DUF420 is not homologous to any known HAS (global mean and median identity 8–13% to different HAS types, Supplementary Table 8), and phylogenetic methods are not suitable to be used with such low identity values (Doolittle, 1981, 1989; Vogt et al., 1995; Rost, 1999; Chang et al., 2008; Schwarz et al., 2010; Meier and Söding, 2015; Habermann, 2016). Thus, its incorporation into the multiple sequence alignment and into the maximum likelihood reconstruction undermines the validity of the phylogeny, and the evolutionary scenario drawn from it should therefore be considered unreliable. Even if DUF420 is a distant homolog, it is conceptually incorrect to include it in phylogenetic reconstructions, as the method fails for identities below 25% (Doolittle, 1981, 1989; Vogt et al., 1995; Rost, 1999; Chang et al., 2008; Schwarz et al., 2010; Meier and Söding, 2015; Habermann, 2016). In the scenario proposed by Degli Esposti et al. (2021), type 0 would represent the ancestral form of HAS, having originated in acidophilic bacteria, which is not supported by our data. Our results indicate that the root separates type 2 from type 1, consistent with previous findings by He et al. (2016), with type 2 possibly having originated within *Pseudomonadota*, and type 1B representing the ancestral form of type 1. This was likely followed by several independent events of cysteine pair loss within the type 1 side, whereas type 2 may have been transferred to the *Bacillota* ancestor and to a few other sequences from diverse affiliations. In our scenario, type 0, instead of being ancestral, would represent a group of fast-evolving enzymes with potential HAS activity (long-branch) that undergo sequential losses of the cysteine pairs. In Archaea, type 1A and potential type 1A^*^ would have originated first by fission and then by re-fusion, from a type 1C enzyme. Although seemingly counterintuitive, this process is supported by the placement of type 1A^*^, which emerges from within the type 1A clade. Alternatively, based on the structural phylogeny, type 1A^*^ may have originated from type 1B sequences. In both scenarios, the type 1A^*^ clade has a statistically low bootstrap support, leaving its exact phylogenetic position unresolved. Given the widespread non-monophyly among types, a simplified and biologically coherent classification may be more appropriate, assuming that such a scheme is justified beyond type 1 and type 2, and potentially type 0 and type 1A.

A keen observer may pose a question as to what makes type 1A^*^ different from type 1C, considering they both contain only one Cys pair and 8 TM helices, as these qualities would group them into the same type by some classifications. There are several features that prompted us to classify these sequences separately. First, type 1A^*^ sequences are taxonomically restricted to *Halobacteria*, while type 1C was not identified in this lineage in our study or any other. Second, the presence of Phe instead of His at the H3 position in the majority of type 1A^*^ sequences creates a difference at the level of conserved residues. Third, type 1A^*^ sequences have a higher mean and median local and global identity to types 1A, 1B, and even 1D than to the type 1C sequences. Finally, it is unclear whether type 1A^*^ sequences are even *bona fide* HAS. In our opinion, only further experimental studies may answer what type 1A^*^ truly is. For now, it made sense to differentiate it from the canonical type 1C to highlight its unique features and draw attention to them, considering this (sub)type of HAS has never been previously described or discussed.

Further divisions into subtypes might mix paralogous with orthologous enzymes under the same classification type, and so far, it is not clear if the Cys-Cys play a structural or catalytic role (2nd pair), and if this influences catalysis at all.

Our results provide additional context for the evolution of aerobic respiration in Eukaryotes. As has been shown in previous works (Esser et al., 2004; Ku et al., 2015; Brueckner and Martin, 2020), eukaryotic genes for energy metabolism and cofactor synthesis are of bacterial origin, and bacterial genes outnumber archaeal genes in eukaryotic genomes. HAS is no exception: as discussed by He et al. (2016), most of the eukaryotic HAS sequences belong to type 2, with a singular known exception, a HAS of type 1B in *Andalucia godoyi*, a biflagellate microorganism isolated from soil and belonging to the deepest branch of Jakobids (Lara et al., 2006). This HAS sequence is thought to be a result of an LGT event from Bacteria. Of note, the frequency of LGT from Bacteria and Archaea to Eukaryotes is likely rare, and remains a matter of ongoing debate (Ku and Martin, 2016; Martin, 2017, 2018; Leger et al., 2018; van Etten and Bhattacharya, 2020; Keeling, 2024), which is beyond the scope of this manuscript. Suffices to say that most eukaryotic HAS sequences cluster within type 2 clade of *Alphaproteobacteria* (except *A. godoyi* sequence, which clusters within type 1B clade of *Alphaproteobacteria*; He et al., 2016), pointing to its acquisition during the endosymbiotic event involving the mitochondrial alphaproteobacterial ancestor (Esser et al., 2004; Roger et al., 2017; or divergent proteobacterial lineages that remain closely related to the sampled *Alphaproteobacteria*—Martijn et al., 2018) by the asgardarchaeal ancestor (Spang et al., 2015, 2018; Liu et al., 2021; Eme et al., 2023; Zhang et al., 2025) of Eukaryotes. Our phylogenies place the origin of both type 1 and type 2 within *Pseudomonadota*, specifically in *Alphaproteobacteria*, further supporting the scenario that HAS was acquired by Eukaryotes during the mitochondrial endosymbiotic event.

Compared with previous studies that focused on smaller datasets, our analysis provides a comprehensive, large-scale perspective and also underlines the usage of cofactor biosynthesis enzymes to study the evolution of the microbial traits that depend on those cofactors (Padalko et al., 2025). By combining large-scale taxonomic screening with phylogenetic and structural approaches, we elucidate both the key features of HAS and the diversity of lineage-specific adaptations. Importantly, the integration of a structural alphabet and structural phylogenetics was essential for resolving relationships that purely sequence-based methods could not clarify, highlighting the utility of structure-informed phylogenetics in enzyme evolution. As with all comparative genomic studies, certain limitations must be acknowledged. Incomplete or fragmented genomes may have led to underrepresentation of HAS in some taxa, and functional inference derived from *in silico* models remains a prediction or indirect inference, requiring experimental validation. Moreover, while we used the canonical comparative genomics cut-off values for homologs of equal or higher than 25% identity and equal or below 10^−10^ E-value, it is possible that a few divergent HAS sequences may not have passed these cut-offs. However, lowering the established cut-offs to try to capture potential false negatives has a trade-off of acquiring more false positives, which can hinder the subsequent analysis. Nevertheless, the extent of the dataset and the convergence of evidence from multiple analytical approaches (motifs, synteny, phylogenies) provide confidence in our main conclusions. Future work should focus on the experimental characterization of divergent HAS types, particularly those from type 0, type 1D, and type 1A^*^.

## Data Availability

The datasets presented in this study can be found in online repositories. The names of the repository/repositories and accession number(s) can be found in the article/Supplementary material.
