# Supplementary material for: Archaeal heme a synthase: evolutionary trajectory distinct from bacterial homologs
Source: Front Microbiol. 2026 Jan 2;16:1706049. doi: 10.3389/fmicb.2025.1706049 (PMC12808453; doi:10.3389/fmicb.2025.1706049)
Supplement: Supplementary file 1 [file Data_Sheet_1.PDF]

Supplementary Material

Archaeal Heme *a* Synthase: Evolutionary Trajectory distinct from Bacterial Homologs

Val Karavaeva[1,2*], Marco Rampin[3], Jordi Zamarreño Beas[1], Lígia M. Saraiva[4], Filipa L. Sousa[1*]

[*]Corresponding authors: val.karavaeva@univie.ac.at, filipa.sousa@univie.ac.at

[1]*Department of Functional and Evolutionary Ecology, University of Vienna, Austria*

[2]*Vienna Doctoral School of Ecology and Evolution, University of Vienna, Austria*

[3]*Division of Environmental Geosciences, Center for Microbiology and Environmental Systems Science, University of Vienna, Austria*

[4]*Instituto de Tecnologia Química e Biológica António Xavier, Universidade Nova de Lisboa, Oeiras, Portugal*

1. Supplementary Figures

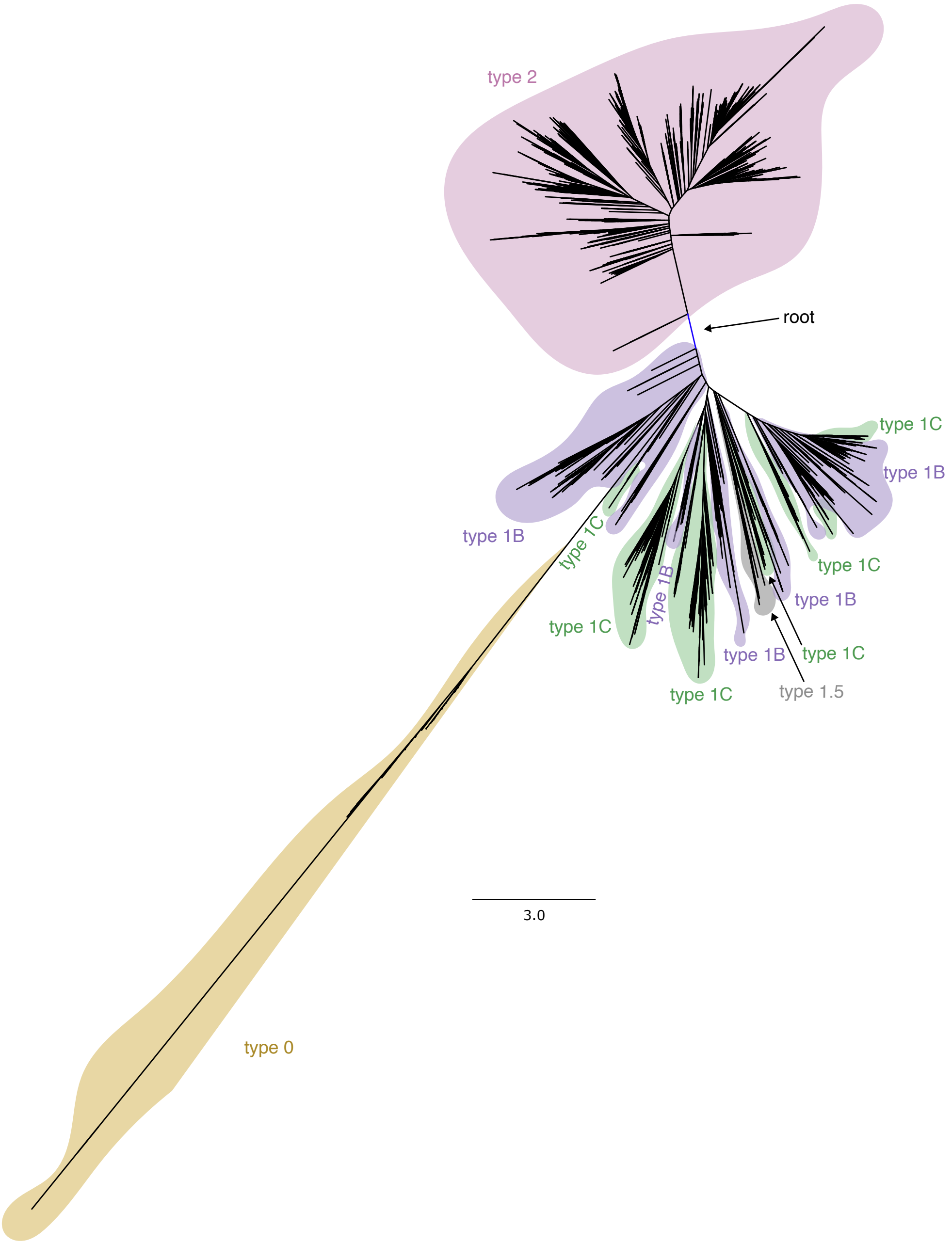

Supplementary Figure 1. *Maximum likelihood phylogenetic reconstruction of heme a synthase without types 1A and 1A*, colored in Fig. 4. Model – Q.Pfam+F+R10.*

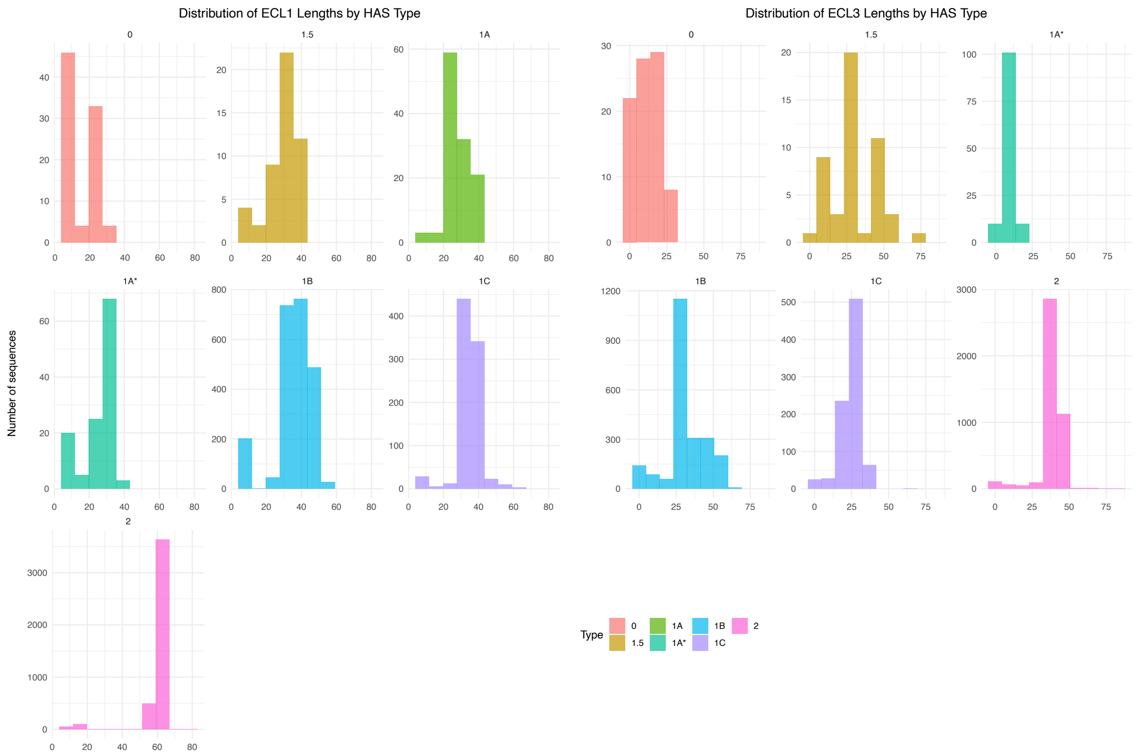

Supplementary Figure 2. *Distribution of number of aminoacids in loop 1 (ECL1) and loop 3(ECL3) per type of HAS. The bar plots represent the number of sequences (y-axis) containing a certain number of aminoacids (x-axis). Of note, type 1A does not contain the ECL3 loop.*

2. Supplementary Table legends

Supplementary Table 1. Query sequences of heme a synthase with taxonomic and genomic information

Supplementary Table 2. Genomic database including taxonomic information, completeness and contamination.

Supplementary Table 3. Sequences added from Degli Esposti et al 2021 with type classification

Supplementary Table 4. Functional annotation of HAS sequences

Supplementary Table 5. Taxonomic distribution of different types of heme a synthase per genome.

Supplementary Table 6. Synteny analysis: Syntenic blocks; counts; and most frequent neighbors

Supplementary table 7. Lengths of extracellular loops ECL1 and ECL3 : Lengths per protein sequence; Mean, median, min and max lengths per HAS type

Supplementary Table 8. HAS Intertype identity: Global identity; Local identity
